# The Role of MR Enterography in Assessing Crohn's Disease Activity and Treatment Response

**DOI:** 10.1155/2016/8168695

**Published:** 2015-12-27

**Authors:** Matthew P. Moy, Jenny Sauk, Michael S. Gee

**Affiliations:** ^1^Division of Abdominal Imaging, Massachusetts General Hospital, Harvard Medical School, Boston, MA, USA; ^2^Division of Pediatric Imaging, Massachusetts General Hospital, Harvard Medical School, Boston, MA, USA; ^3^Division of Gastroenterology, Massachusetts General Hospital, Harvard Medical School, Boston, MA, USA

## Abstract

MR enterography (MRE) has become the primary imaging modality in the assessment of Crohn's disease (CD) in both children and adults at many institutions in the United States and worldwide, primarily due to its noninvasiveness, superior soft tissue contrast, and lack of ionizing radiation. MRE technique includes distention of the small bowel with oral contrast media with the acquisition of T2-weighted, balanced steady-state free precession, and multiphase T1-weighted fat suppressed gadolinium contrast-enhanced sequences. With the introduction of molecule-targeted biologic agents into the clinical setting for CD and their potential to reverse the inflammatory process, MRE is increasingly utilized to evaluate disease activity and response to therapy as an imaging complement to clinical indices or optical endoscopy. New and emerging MRE techniques, such as diffusion-weighted imaging (DWI), magnetization transfer, ultrasmall superparamagnetic iron oxide- (USPIO-) enhanced MRI, and PET-MR, offer the potential for an expanded role of MRI in detecting occult disease activity, evaluating early treatment response/resistance, and differentiating inflammatory from fibrotic strictures. Familiarity with MR enterography is essential for radiologists and gastroenterologists as the technique evolves and is further incorporated into the clinical management of CD.

## 1. Introduction

Crohn's disease (CD) is a chronic inflammatory disorder that can occur throughout the gastrointestinal tract and is characterized by episodes of relapse and remission. The annual incidence of CD is highest in North America (20.2 per 100,000 person-years) and the highest prevalence of CD is in Europe (322 per 100,000 person-years) [1]. However, CD is emerging in the developing world correlating with rises in industrialization and westernization [2]. CD has a peak incidence in the second and third decades of life with 25% of all cases presenting in childhood or adolescence [3]. CD begins as an inflammatory process affecting various portions of the gastrointestinal tract (most commonly the terminal ileum) and often leads to progressive irreversible bowel damage [4]. CD complications include stricturing disease associated with symptoms related to bowel obstruction and penetrating disease associated with abscess and fistula formation [5]. As CD is a chronic condition that can affect different segments of the gastrointestinal tract over time, anti-inflammatory medical therapy is the primary treatment strategy. Surgical bowel resection is typically reserved for CD patients with stricturing or penetrating disease refractory to medical therapy.

The role of imaging in patients with CD arose from the need to evaluate portions of small bowel inaccessible to optical endoscopy. Barium fluoroscopic methods such as enteroclysis and small bowel series historically have been used to evaluate the small bowel and demonstrate characteristic features associated with CD. However, these modalities are limited in their ability to evaluate extraluminal and extraintestinal disease manifestations and to evaluate acutely ill patients. CT enterography (CTE) is a specific cross-sectional imaging technique that is tailored to evaluate the small bowel, through the use of large volume neutral oral contrast and image acquisition in the enteric phase of intravenous contrast enhancement [6]. Because of its broad availability in emergency rooms, rapid image acquisition, and ability to evaluate mural, extraluminal, and extraintestinal CD manifestations, CTE has become a standard imaging tool for CD evaluation [7, 8]. However, in recent years, attention has been focused on the potential ionizing radiation risks associated with CT scans, particularly in the CD population that likely requires multiple imaging studies over the course of their disease [9, 10]. A meta-analysis showed that up to 10% of CD patients have had exposure to ≥50 millisieverts (mSv) of ionizing radiation exposure from imaging studies (mostly due to CT scans), a threshold above which a nonzero radiation risk has been suggested [11].

MR enterography (MRE) developed as an alternative imaging technique to CTE for small bowel imaging and, in many institutions, has largely replaced CTE as the primary cross-sectional imaging modality for both adult and pediatric patients with CD [12–15]. MRE does not utilize ionizing radiation and allows the bowel to be imaged at multiple time points, enabling the acquisition of cinematic images to evaluate peristalsis and dynamic contrast-enhanced images to characterize mural enhancement. These techniques allow MRE to provide both anatomic and functional information. MRE also provides superior soft tissue contrast resolution compared to CT and can help to characterize bowel wall tissue composition [16]. As CD affects the small bowel in at least 70% of patients, cross-sectional imaging such as CTE and MRE can help the clinician evaluate areas of the small bowel that cannot be accessed by standard ileocolonoscopy, rule out complications such as strictures and abscesses requiring urgent intervention, and also assess disease activity [17].

## 2. MRE Technique

MRE combines large volume oral contrast distention of the bowel with T2-weighted, balanced steady-state free precession, and multiphase T1-weighted fat-suppressed contrast-enhanced sequences to optimize detection of abnormalities in the bowel wall. A multichannel phased array body coil is used, with imaging field of view extending from the top of the transverse colon to the bottom of the anal sphincter complex. MRE is performed on both 1.5T and 3T clinical scanners, with the higher 3T field strength being advantageous for higher signal to noise ratio and faster scan times but at a cost of higher susceptibility artifacts from air within bowel. Oral contrast preparations vary among different institutions but typically are biphasic, demonstrating low signal intensity on T1-weighted sequences to visualize bowel wall enhancement and high signal intensity on T2-weighted sequences to visualize bowel wall thickening. Some common biphasic oral contrast agents include water, polyethylene glycol, sorbitol, mannitol, dilute barium sulfate, and locust bean gum [18, 19]. Nonabsorbable enteric contrast agents are helpful to maintain bowel distention throughout the duration of the study. The total volume of enteric contrast needed to distend the small bowel in adults ranges from 1 to 2 L administered over 45–60 minutes, with a lower volume given to pediatric patients based on weight [6, 20, 21]. Some institutions administer glucagon or hyoscine butylbromide as a bowel paralytic to reduce peristalsis and resultant motion artifact, although this can cause nausea in some patients [19, 22–27]. Patient positioning also varies by institution, with many institutions favoring MRE performed in the supine position for patient comfort and others preferring prone position positioning to compress the bowel and decrease scan times.

Typical MRE pulse sequences include single-shot T2-weighted images and balanced steady-state free precession (bSSFP) sequences in the coronal plane to provide motion-free assessment of the bowel wall, mesentery, and extraintestinal regions; cinematic thick slab coronal bSSFP images to evaluate peristalsis and distinguish underdistended from inflamed bowel loops; axial T2-weighted fat-suppressed images to assess for bowel well edema and intra-abdominal fluid collections; coronal multiphase 3D T1-weighted fat-suppressed postcontrast images to evaluate bowel wall enhancement and mesenteric vascularity; and delayed axial T1-weighted fat-suppressed images to evaluate for penetrating disease complications including fistulae and abscesses. Diffusion-weighted imaging (DWI) may also be performed with *b* values of 0–800 s/mm^2^ (usually acquired with a low *b* value of 0–50 s/mm^2^ and 1-2 additional higher values) to aid in detection of bowel wall inflammation and extraluminal collections.

At our institution, the MRE protocol includes a multichannel body phased array coil with patents scanned in the supine position. Our oral contrast preparation is 1350 mL of dilute barium and sorbitol (VoLumen E-Z EM, Lake Success, NY) administered over 45 minutes prior to the examination. For pediatric patients and adults who cannot tolerate the oral contrast agent, polyethylene glycol (PEG; Miralax) is administered as four (or fewer based on patient weight) aliquots of 17 g PEG diluted in 12 ounces of water with sugar-free fruit flavoring administered over the same time period. Our standard MRE pulse sequences are summarized in Table 1. All of the sequences are performed with breath-hold technique except for the DWI sequence. Postcontrast sequences are performed after the administration of intravenous gadopentetate dimeglumine (Magnevist; Bayer).

## 3. Current Role of MRE in Clinical Management of CD Patients

MRE currently is used in a variety of clinical scenarios, including (1) evaluating distribution of disease at the time of initial CD diagnosis, (2) assessing disease activity in CD patients during symptomatic recurrence, (3) assessing and tracking progression of extraintestinal CD manifestations, and (4) evaluating CD patients with stricturing disease to distinguish inflammatory from fibrotic stenoses.

### 3.1. Defining Extent of Disease

Disease extent is important to determine as patients with distal ileal disease on colonoscopy can have more proximal disease that is not accessible to endoscopic visualization. CD involvement of the proximal small bowel is important to recognize because it can be associated with symptoms related to malabsorption (vitamin deficiencies, weight loss, and steatorrhea) and has a higher risk of stricturing behavior and requirement for multiple surgeries [28]. In addition, precise delineation of the length of small bowel involvement in the decision whether to perform surgical resection of bowel is refractory to medical therapy, balancing the potential for symptomatic relief against the risk of short gut syndrome [29]. In a study by Samuel et al., 54% (*n* = 67) with normal terminal ileal mucosa on colonoscopy had evidence of significant inflammation on CTE. Roughly one-third of patients had evidence of complications (penetrating or stricturing) or extraluminal manifestations that were not previously appreciated [30]. Studies comparing the ability of MRE, small intestine contrast US (SICUS), and capsule endoscopy (CE) to evaluate the small bowel in pediatric CD found that in the proximal small bowel the sensitivity of all three modalities was similar but the specificity of MRE was significantly higher than that of CE (61%). A recent paper comparing MRE to reference video capsule and optical endoscopy demonstrated sensitivity and specificity values ranging from 70 to 100% for detection of active disease throughout all segments of small and large bowel [31].

### 3.2. Assessment of Disease Activity

**Figure 1:**
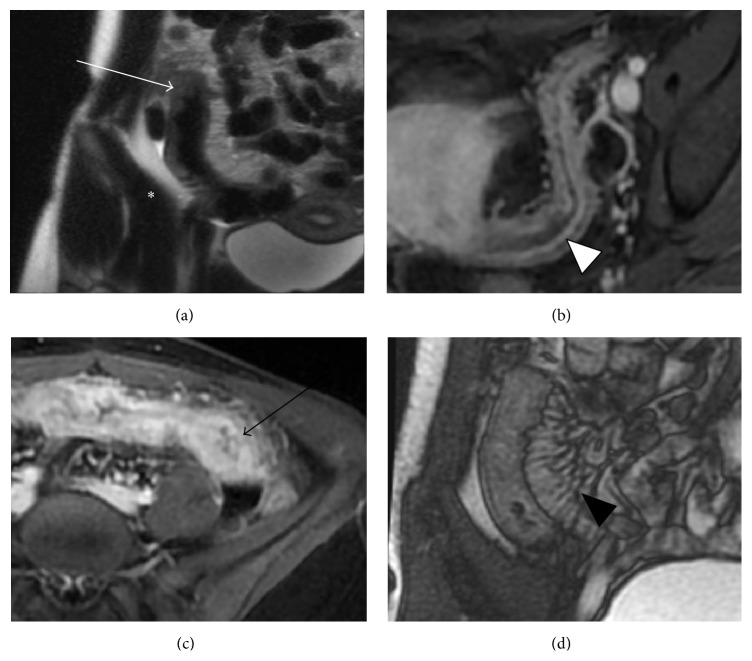
MR enterography features of active Crohn's disease. (a) Coronal T2-weighted image demonstrates wall thickening (arrow), axial T1-weighted fat-suppressed postcontrast images obtained in enteric (b) and delayed (c) phases demonstrate early mucosal ((b), arrowhead) with progressive transmural ((c), arrow) enhancement; coronal balanced steady-state free precession image (d) demonstrates mesenteric hypervascularity (arrowhead).

A number of MRE imaging features (Figure 1) have been validated as biomarkers of active Crohn's disease compared with clinical, endoscopic, and histological reference. Bowel wall thickening, in both the small bowel and colon, has been extensively studied and validated as a sign of active inflammation [23, 24, 32–40]. Many authors have considered the bowel to be abnormal with a wall thickness of greater than 3 mm [32, 34, 37], and increasing bowel wall thickness correlates with increasing severity of disease. Bowel wall edema, indicated by mural hyperintense signal compared with skeletal muscle on T2-weighted sequences, is another indicator of active inflammation [23, 37, 39–42]. Mural T2 hyperintense signal is often best appreciated on fat-saturation sequences [27, 35, 43]. The degree and pattern of bowel wall enhancement are also associated with disease activity. While diffusely increased mural enhancement compared to normal bowel often corresponds to active inflammation, it is less specific than other imaging features and can be observed in normal underdistended bowel loops [23, 34, 37–39, 42, 44]. Early mucosal hyperenhancement during the enteric phase has been shown to be a more specific sign of active inflammation and correlates with mucosal neutrophilic infiltration [32, 45, 46]. A stratified enhancement pattern (hyperenhancement of the mucosa/submucosa complex and serosa with an intervening hypoenhancing muscularis propria) has been observed with both active inflammation and intestinal fibrosis [35, 45, 47]. Quantitative analyses of enhancement kinetics have also been shown to be effective predictors of active inflammation but are not implemented in routine clinical practice [37, 45]. Mucosal ulceration is an uncommon MRE finding of active disease in CD and requires adequate distention of the small bowel for reliable detection. When compared with endoscopic evidence of inflammation, Oussalah et al. found that mucosal ulceration detected at MRE had a sensitivity of 37.5%, specificity of 88.79%, and AUC of 0.631 (*p* = 0.0001) [48]. Mucosal ulceration on MRE is usually seen in more severe cases of inflammation, likely due to the generally poor distensibility of actively inflamed bowel loops limiting ulcer conspicuity [23, 49].

Several extramural mesenteric MR signs of active disease have also been described, though their performance has been variable in the literature. Perimural T2 hyperintensity, corresponding to adjacent fatty edema and inflammation, correlates with active inflammation and can appear as a small rim of fluid in severe cases [39, 41]. The comb sign, or mesenteric hypervascularity characterized by dilated, tortuous, and conspicuous prominence of the vasa recta supplying an inflamed loop of bowel, was first described as a CT finding [50] of active inflammation in Crohn's disease but has also been shown to correlate with active inflammation on MR imaging [38, 51]. Mesenteric lymphadenopathy, characterized by size greater than 1 cm short axis [23] or increased enhancement [33], is an insensitive sign of active inflammation and can be present adjacent to bowel loops with a history of inflammation. These extramural findings are associated with active inflammation but are not consistently present and are best used as supportive evidence in addition to mucosal or mural abnormalities.

### 3.3. Distinguishing Inflammatory from Fibrotic Strictures

A common complication of longstanding Crohn's disease is the development of strictures that produce symptoms including nausea, vomiting, and bowel obstruction [5]. The distinction between inflammatory and fibrotic strictures is important clinically because of its impact on clinical decision making [16, 46]. Inflammatory strictures are due to acute transmural inflammation and edema and are typically treated with anti-inflammatory medications. In contrast, fibrotic strictures are caused by chronic mural deposition of extracellular matrix proteins and are treated with mechanical therapies consisting of surgical resection or endoscopic dilation. MRE has been shown in a number of studies to be helpful in distinguishing the two types of strictures. MRE features associated with intestinal fibrosis include wall thickening, T2 hypointense signal in comparison to skeletal muscle, and minimal (no more than mild) mural enhancement on multiphase postcontrast images [46, 52]. One European study suggested that fibrotic lesions on MRE could be confirmed histologically with an MRE sensitivity of 95.8%, a specificity of 100%, and a diagnostic accuracy of 97.9% [53]. However, recent studies suggest that fibrosis and active inflammation often coexist in strictured bowel, and superimposed active disease can obscure underlying fibrosis on imaging [38, 46, 54]. One study found that MRE correlated well with histological evidence of fibrosis in the absence of superimposed acute inflammation, (83% accuracy/83% sensitivity/83% specificity); however, MRE performance decreased for bowel segments with concomitant active inflammation/fibrosis [46]. A prospective study comparing MRE and CTE for detection of fibrosis in young CD patients compared with histologic reference demonstrated MRE to have higher accuracy and sensitivity [16].

## 4. The Emerging Role of MRE in Evaluation of CD Treatment Response

Assessing CD response to therapy is challenging because clinical symptoms, which typically are the basis for CD patients to undergo medical evaluation for disease exacerbation, often do not correlate with objective measures of disease activity [55, 56]. Clinical indices such as the Crohn's Disease Activity Index (CDAI) and serum laboratory markers of inflammation such as erythrocyte sedimentation rate (ESR) and C-reactive protein (CRP) provide more quantitative assessment of activity but are imperfect biomarkers because of their susceptibility to non-Crohn's related inflammation and inability to resolve inflammatory changes at the individual bowel segmental level. Currently, endoscopic evaluation is considered the gold standard for CD treatment response assessment. Advantages include more objective assessment of disease severity, ability to follow individual bowel segments over time for changes in disease severity, and ability to perform endoscopic mucosal biopsy to obtain microscopic evaluation of disease. Two of the mostly widely used endoscopic indices are Crohn's Disease Endoscopic Index of Severity (CDEIS) and the Simple Endoscopic Score for Crohn's Disease (SES-CD). Both of these have significant limitations including high complexity precluding routine clinical use, interuser variation in assessment, and lack of established cutoff values to define response/remission [57].

Recent evidence suggests that endoscopic mucosal healing, defined as resolution of visible mucosal inflammatory changes in areas of prior inflammation, may be an important therapeutic endpoint. The concept of mucosal healing as a therapeutic endpoint derives from the observation that endoscopic lesions often precede the onset of clinical symptoms by months or even years [57]. By inference, treating to resolution of endoscopically apparent lesions should be associated with durable clinical response. In clinical studies, mucosal healing has been shown to be an independent indicator of sustained clinical remission and is associated with reduced rates of hospitalization and surgery in CD patients undergoing medical therapy [58–66]. Consequently, mucosal healing has become a therapeutic target of treatment algorithms in both adults and children and is an endpoint of several CD clinical trials [60–62, 67–69].

While endoscopic assessment is the current gold standard for evaluating response to CD therapy in clinical trials, serial endoscopy to evaluate treatment response is not typically performed in routine clinical practice because of procedure invasiveness. As a result, endoscopy in the posttreatment setting is typically reserved for evaluating disease activity in patients who are clinically symptomatic. Because of its noninvasiveness, MRE has great potential for assessing therapeutic response and would be a valuable biomarker to assess treatment efficacy early following initiation. This could detect therapeutic resistance prior to clinical exacerbation of symptoms and provide a temporal window for dose escalation or addition of combination therapy to maintain clinical remission. Furthermore, because drugs such as TNF*α* antagonists are expensive and carry potential long-term risks of serious infection or malignancy, MRE is likely to help minimize unnecessary financial cost and long-term toxicities in nonresponders [70].

**Figure 2:**
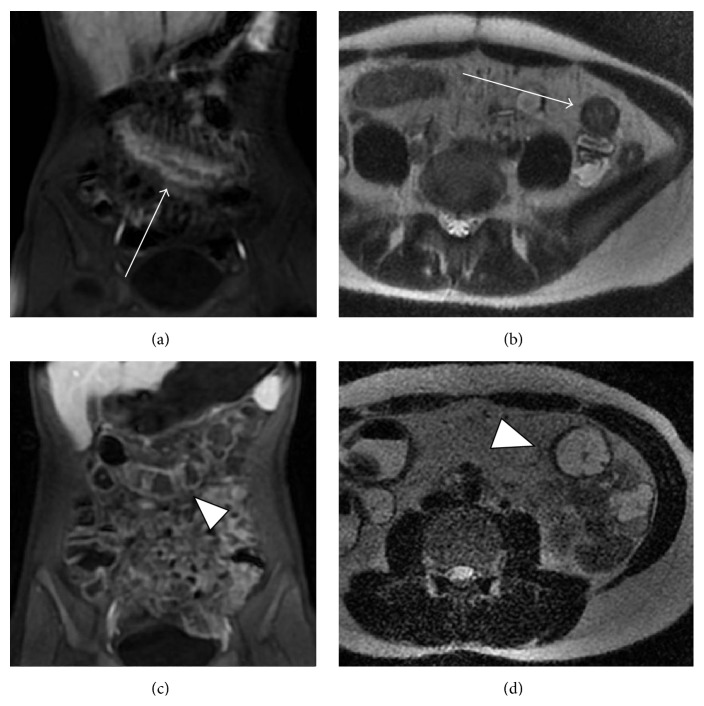
Mucosal healing on serial MRE in a patient with Crohn's disease affecting the transverse colon. (a) Coronal T1-weighted postgadolinium with fat saturation and (b) axial T2-weighted images demonstrating bowel wall thickening, mural edema, mucosal hyperenhancement, and the comb sign (arrows) on initial imaging. Subsequent MRE performed after treatment ((c) and (d)) demonstrates normalization of imaging findings (arrowheads) correlating with mucosal healing at endoscopy.

Recent studies suggested that MRE is able to detect mucosal healing. In one prospective, multicenter study of 48 patients with CD with active disease and ulcers in at least one ileocolonic segment, all patients underwent ileocolonoscopy and MRE at baseline and 12 weeks after completing treatment with TNF*α* antagonists or corticosteroids. MRE had 90% accuracy for reporting ulcer healing and 84% accuracy for evaluating endoscopic remission [71]. Furthermore, the degree of change in the endoscopic CDEIS scores correlated with MRE features. MRE findings of mucosal healing on serial imaging are depicted in Figure 2. Another prospective study found persistent transmural inflammation on MRE in most patients after treatment with TNF*α* antagonists, despite improvements seen on MRE as early as 2 weeks [72]. The significance of persistent transmural disease in the setting of endoscopic healing is unclear and suggests that MRE may be providing additional information on the CD inflammatory process beyond the mucosal changes visualized by endoscopy [4].

## 5. MRE Indices of Disease Activity

Several attempts have been made to establish and validate standardized MRE criteria for disease activity in CD for clinical use and as a potential endpoint of clinical trials. The most widely studied index of activity based on MRE is the magnetic resonance index of activity (MaRIA). Rimola et al. used ileocolonoscopy as a reference standard to develop and validate an index of activity for MRE based on various imaging features. Bowel wall thickness, mural edema, mucosal ulceration, and relative contrast enhancement (RCE) were found to correspond to endoscopic evidence of inflammation (*p* ≤ 0.001). A subsequent regression model using these imaging features generated the MaRIA score: 1.5 × bowel wall thickness in mm + 0.02 × RCE + 5 × edema + 10 × ulceration. The authors found a strong correlation between MaRIA and the validated endoscopic score of disease activity, Crohn's Disease Endoscopic Index of Severity (CDEIS), both for individual segments and when summed for a global score [23, 71, 73, 74].

Another MRE index known as Crohn's disease activity score (CDAS) was derived by Steward et al. retrospectively using the acute inflammation score (AIS), a histopathological grading system, as reference. Multivariate analysis was performed on multiple quantitative and qualitative MR features of active disease which had a significant association with AIS yielded the final CDAS score: 1.79 + 1.34 × mural thickness + 0.94 × mural T2 score. A cutoff of 4.1 for a single bowel segment yielded a sensitivity of 81% for the detection of histopathological inflammation, specificity of 70%, and AUC of 0.76 [39]. A recent study from 2014 modified the CDAS into a global assessment score called the MRE global score (MEGS), which is a sum of scores of qualitative and semiquantitative grading of bowel wall thickness, mural T2 signal, degree and pattern of contrast enhancement, length of the diseased segment, and extramural inflammatory features. Makayanga et al. prospectively enrolled and evaluated patients with CD by MRE, comparing MEGS with laboratory and clinical biomarkers of activity. The final MEGS logistical regression compared with fecal calprotectin >100 *μ*g/g yielded the formula *α* = 1.8 × wall thickness + 0.08 × mural T2 + 0.19 × length − 0.192, where *α* is the natural logarithm of the probability of that patient having active disease defined by elevated fecal calprotectin. The MEGS model had a sensitivity of 65% and a specificity of 78% for the detection of active disease [40].

## 6. New and Emerging MRE Biomarkers for Crohn's Disease

### 6.1. Diffusion-Weighted Imaging (DWI)

**Figure 3:**
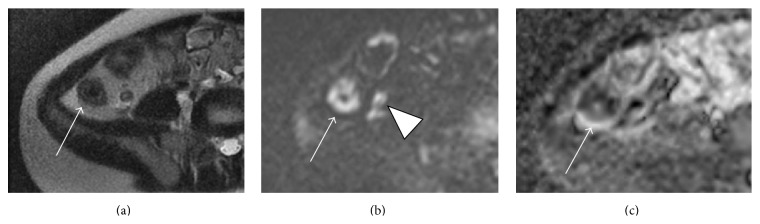
Diffusion-weighted imaging of active Crohn's disease. (a) Axial single-shot T2-weighted image demonstrating wall thickening and edema in the terminal ileum; (b) DWI and (c) ADC images (*b* = 600) demonstrating hyperintense signal on DWI and low ADC (arrow) in the same region of the terminal ileum indicating restriction of diffusion. There is also restricted diffusion in the distal ileum and appendix (arrowheads) consistent with additional areas of inflammation.

DWI is becoming more widespread in abdominopelvic imaging, including imaging in Crohn's disease. At our institution, axial echo planar DWI sequences are a standard part of the MRE protocol in both children and adults. Restricted microscopic diffusion of water molecules, manifesting as high signal intensity on high *b* value (typically in the 400–800 range) diffusion-weighted sequences and corresponding low signal intensity on derivative apparent diffusion coefficient (ADC) maps, has been observed in the bowel of patients (Figure 3) with active Crohn's inflammation and is thought to be related to the immune cell infiltration accompanying microscopic inflammation [75, 76]. It is also important to recognize that DWI technique can affect quantitative measurements as the use of different *b* values can result in slightly different ADC calculations [77].

Multiple studies have demonstrated significant reduction in bowel wall ADC values in actively inflamed segments compared with either standard MRE pulse sequence or histologic reference [26, 37, 75–79]. Freiman et al. demonstrated that fast diffusion restriction, which reflects the bulk motion of intravascular molecules in microcapillaries, contributes more to the reduction in ADC values in areas of active inflammation than slow diffusion, which is associated with the Brownian motion of water molecules [77]. This finding is consistent with previous studies showing a change in microvascular volume in bowel segments with active inflammation [80–82] and suggests that the DWI changes accompanying active CD may be more related to alteration in vascularity or vascular permeability rather than immune infiltration. The Clermont score is an MR index similar to MaRIA for assessing CD activity on noncontrast MRE with ADC values substituting for relative contrast enhancement. The Clermont score was derived using a multivariate linear regression model, generating the formula −1.321 × ADC  (mm^2^/s) + 1.646 × WT (mm) + 8.306 × ulceration + 5.613 × edema + 5.039, which was shown to strongly correlate with MaRIA [26, 83]. However, the Clermont score has not yet been validated against endoscopy or histology as a reference standard.

At this time the level of evidence suggests that DWI may be a useful adjunct in the evaluation of CD activity, but its added value over standard MRE including multiphase postcontrast images has not yet been demonstrated. Further prospective studies comparing noncontrast abdominopelvic MRI with DWI with contrast-enhanced MRI are needed to validate its use in lieu of intravenous gadolinium contrast. DWI is likely to be of benefit in CD patients who cannot receive intravenous contrast, such as patients with renal insufficiency or young children being scanned awake without intravenous cannulation.

### 6.2. Magnetization Transfer MR Imaging of Fibrosis

Magnetization transfer (MT) is an MR technique which generates image contrast between protons in free water molecules and those within water molecules associated with large molecules, such as collagen. The MT effect is greater in the latter situation, potentially serving as an indirect measure of the relative concentration of these macromolecules. MT pulse sequences include 2D or 3D gradient echo sequences performed before and after the addition of an off-resonance MT prepulse. The off-resonance pulse saturates the signal of the low-mobility water protons, resulting in a high MT ratio (MTR) in tissues containing high concentrations of macromolecules [84–87]. In the setting of CD, MT is of interest for its potential to detect bowel wall collagen deposition as a marker of intestinal fibrosis. Studies in a rat model demonstrated a higher MT ratio (MTR) in bowel segments with fibrosis (determined by histology) compared with normal bowel segments. There was also a lower T2 signal intensity ratio-to-MTR in bowel segments with fibrosis compared with normal bowel [84, 85]. Similar results were demonstrated in a small study in human patients with CD (9 patients total), with high MTR corresponding to small bowel segments with conventional MRE evidence of fibrostenosing disease [86]. One limitation of clinical fibrosis imaging studies is the availability of full bowel wall thickness histology reference for collagen deposition, as fibrosis occurs in the submucosal and serosal bowel layers and is not seen on endoscopic biopsy. Initial results of MT imaging are promising as a potential discriminator of inflammatory and fibrosing stenosis in CD, though robust human studies with full-thickness histologic validation are lacking at this time.

### 6.3. USPIO-Enhanced MRI

Ultrasmall superparamagnetic iron oxide (USPIO) nanoparticles are a group of targeted MRI contrast agents with dual capability of imaging tissue vascularity and immune cell infiltration. They consist of an iron oxide crystalline core with a dextran-based surface coating [88] and, when administered intravenously, these particles are initially localized to the blood pool and are subsequently taken up by macrophages and target sites of cellular inflammation. USPIO nanoparticles are preferentially phagocytosed by macrophages of the mononuclear phagocyte system, resulting in accumulation in areas of macrophage activity, including inflammation and infection [89–92]. One of these nanoparticles (ferumoxytol) has recently been approved by the FDA for intravenous administration to patients with iron deficiency anemia, and its MR imaging signal characteristics within tissues have been studied. Ferumoxytol-enhanced MRI has been studied in the imaging of inflammation in both animal models [90, 93–97] and in humans, including imaging of myocardial inflammation [96, 98–100], atherosclerosis [92, 101, 102], and neuroinflammation [97, 103–105]. USPIO nanoparticles have been shown to be sensitive for detecting subtle inflammatory activity that is below the resolution of conventional MRI [106, 107]. USPIO accumulation within tissues decreases T2^*∗*^ relaxation time, resulting in decreased signal intensity on T2^*∗*^-weighted images, which can be assessed qualitatively or quantitatively [108, 109].

**Figure 4:**
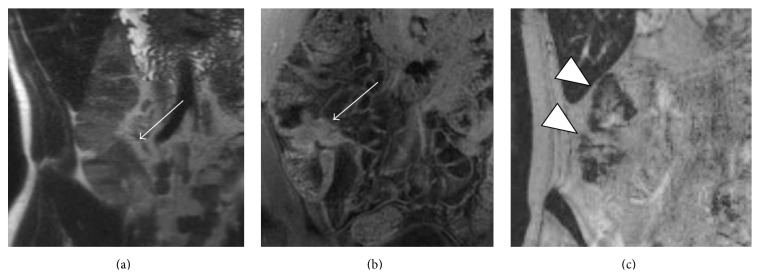
USPIO-enhanced MR imaging of the bowel in a patient with Crohn's disease who received ferumoxytol infusion 24 hours earlier as iron replacement therapy. (a) Coronal T2-weighted and (b) coronal T1-weighted fat-saturated postgadolinium images demonstrating wall thickening and hyperenhancement of the terminal ileum (arrows). (c) Coronal T2^*∗*^-weighted image depicts nanoparticle accumulation in the wall of the cecum and ascending colon (low signal indicated by the arrowheads) indicating inflammatory involvement not visible on conventional MR sequences. Images courtesy of Mukesh Harisinghani, MD.

While there are no published studies yet investigating the role of ferumoxytol-enhanced MRE in CD, T2^*∗*^-weighted imaging of the bowel during the immediate blood pool and delayed macrophage phases could prove to be useful in detecting and quantifying inflammatory activity (Figure 4). Ferumoxytol also offers potential advantages over gadolinium based contrast agents for imaging patients with chronic renal insufficiency. Administration of ferumoxytol is safe in adult and pediatric patients with chronic renal insufficiency without any known links to nephrotoxicity or adverse reactions specific to underlying renal insufficiency (such as nephrogenic systemic fibrosis). Additionally, hemodialysis is not required after its administration [110–113].

### 6.4. ^18^F FDG PET-MRE

18-Fluorodeoxyglucose (^18^F-FDG) positron emission tomography (PET) has been shown to correlate with areas of active inflammation in CD in both children and adults, including the case when it is performed in combination with CT and CTE [114–117]. ^18^F-FDG is transported across the cell membrane via GLUT1 and GLUT3 glucose transporters, preferentially accumulating in cells with high glucose uptake and utilization, which includes inflammatory cells. Once transported across the cell membrane, ^18^F-FDG cannot undergo glycolysis and is trapped within the inflammatory cells, localizing radiotracer uptake to areas of inflammation [118]. This provides additional functional data beyond the structural abnormalities seen with active CD on CT and MRI. In a prospective study of PET-CT in CD patients, Groshar et al. found that bowel segments with CT evidence of active inflammation demonstrated higher FDG standardized uptake values (SUV) compared with normal bowel segments, which also correlated with bowel wall thickening and mural hyperenhancement [114]. Another study found that maximum and mean ^18^F-FDG SUV correlated with endoscopic CDEIS [117], again demonstrating the ability of PET to identify areas of active bowel inflammation.

**Figure 5:**
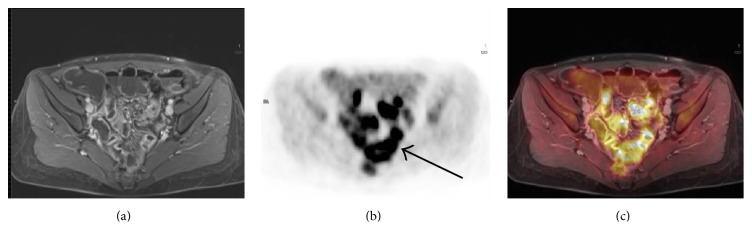
Simultaneous ^18^F-FDG PET/MR in a patient with known Crohn's disease. (a) Axial T1-weighted fat-saturated postcontrast images with mucosal hyperenhancement of several loops of small bowel in the lower abdomen consistent with active inflammation. (b) Attenuation-corrected PET images demonstrate intense ^18^F-FDG uptake in these bowel loops. (c) Fusion overlay images demonstrate localization of ^18^F-FDG avidity to the enhancing small bowel loops. Images courtesy of Onofrio Catalano, MD.

^18^F-FDG PET-MRE performed on hybrid PET-MRI systems that can perform simultaneous or sequential acquisition of PET and MR images could prove to be a valuable addition to standard MRE protocols in the evaluation of active disease in CD. Such hybrid systems have been studied in other inflammatory conditions such as coronary artery disease [119–121]. A particular advantage of simultaneous PET-MRI hybrid imaging is improved coregistration of ^18^F-FDG uptake upon anatomic images of the bowel [122]. In the future, PET-MRI may also incorporate novel PET radiotracers that target inflammatory cytokines or fibrosis. ^18^F-FDG PET-MRE would combine the standard MRE features of disease (e.g., T2 signal intensity and mural hyperenhancement) with quantitative biomarkers (e.g., ADC and SUV) to potentially provide more precise assessment of changes in disease activity over time (Figure 5). PET-MRI is also associated with significantly reduced ionizing radiation exposure to patients compared with PET-CT [123], an important consideration for young CD patients who may require serial imaging over their lifetime. Evidence establishing a clear benefit to the addition of ^18^F-FDG PET to cross-sectional imaging such as MRE in the evaluation of CD is needed to justify the additional patient radiation exposure above conventional MRE alone.

## 7. Conclusion

Crohn's disease is a chronic relapsing and remitting inflammatory disease resulting in progressive bowel damage over time. CD therapy is aimed at suppressing bowel inflammation in order to improve long-term outcomes. Emerging data suggest that mucosal healing or even resolution of transmural inflammation is more appropriate therapeutic goal than symptomatic control alone. Imaging, and MRE in particular, has become central in the treatment algorithm of CD as a noninvasive measure of disease activity and distribution, extraluminal and extraintestinal complications, and treatment response. Changes in disease activity as measured on serial MRE examinations are important for the optimization of treatment protocols and guidance of therapeutic decisions.

MRE is an ideal imaging modality in the assessment of CD, offering both structural and functional information without the use of ionizing radiation. Standardized MRE-based activity indices have been developed and continue to be investigated as objective endpoints of treatment and clinical trials. Developing MR-based techniques, such as DWI, magnetization transfer, USPIO-enhanced MRI, and PET-MR, have the potential to improve the ability of MRE to image the microscopic changes accompanying early inflammation and fibrosis. Familiarity with MR enterography is essential for radiologists and gastroenterologists who participate in the clinical management of CD patients.

## Figures and Tables

**Table 1:** MR enterography pulse sequences.

Sequence	Trade name	Imaging plane
Single-shot T2	SSFSE/HASTE	Axial, coronal
Balanced steady-state free procession (bSSFP)	TrueFISP/FIESTA	Coronal
Fat-saturated single-shot T2	SSFSE/HASTE	Axial
3D cinematic bSSFP		Coronal
Diffusion-weighted (DWI)		Axial
3D T1 postcontrast fat-saturated gradient recalled echo at 45 s, 70 s, and 180 s	VIBE/LAVA	Coronal
Delayed 3D T1 postcontrast fat-saturated gradient recalled echo	VIBE/LAVA	Axial
